# Practices and Perceptions of Animal Contact and Associated Health Outcomes in Pregnant Women and New Mothers

**DOI:** 10.3389/fvets.2016.00005

**Published:** 2016-02-01

**Authors:** Hsin-Yi Weng, Kimberly Ankrom

**Affiliations:** ^1^Department of Comparative Pathobiology, Purdue University, West Lafayette, IN, USA

**Keywords:** pregnancy, infant, injury, zoonoses, companion animal, human–animal interaction

## Abstract

Companion animals play an important role in our society. However, pregnant women and new mothers might have specific concerns about animal-associated health outcomes because of their altered immune function and posture as well as their newborn babies. The study was conducted to collect baseline data for developing an evidence-based intervention for pregnant women and new mothers to help them adopt certain behaviors to prevent adverse animal-associated health outcomes. A survey, using the Health Belief Model as the theoretical framework, was developed and administered to 326 women attending the Women, Infants, and Children programs in Illinois and Indiana in 2015. Prevalence of dog and cat ownership was estimated to be 39% (95% CI: 33–45%) and 26% (95% CI: 21–31%), respectively. Regardless of pet ownership, 74% of the respondents reported having some type of animal contact in the past month. Pregnancy or the birth of a child altered some animal contact practices among the study participants; particularly a discontinuation or decrease in cleaning cat litter boxes. Reports of diseases contracted from animals were low (4%) in this study. By contrast, animal-associated injuries were prevalent (42%), and the majority were caused by animals the respondents owned (56%). Overall, respondents indicated that they appreciated the benefits of a program addressing animal-associated health outcomes and did not indicate strong resistance to adopt certain behaviors. The majority recognized human health-care providers as a source of information about animal contact and associated health outcomes but less frequently identified veterinarians as a source for such information. In addition, although most of the respondents felt that health-care providers and veterinarians should initiate discussions about preventing animal-associated illness and injuries, only 41% among those who had visited doctors or prenatal care services reported that their health-care providers discussed these issues with them. The results indicate the importance of an intervention concerning animal contact and associated health outcomes for the target population and provide insights to the potential implications of program implementation.

## Introduction

Companion animals play an important role in our society. According to the 2012 United States pet ownership survey by the American Veterinary Medical Association (AVMA), 37% of households in the United States own dogs and 30% own cats, and that the total number of pet dogs and cats is approximately 144 million (1). Furthermore, 63% of the surveyed owners consider their pets to be family members, showing a strong bond between companion animals and their human counterparts. Pregnant women and new mothers, however, might have specific concerns about animal-associated health outcomes. Pregnancy has various effects on the female body, including changes to immune function and posture, such as weight gain, shifting posture, and changes in the sense of balance (2). Pregnant women, therefore, might be at greater risk of contracting zoonotic diseases and experiencing other adverse health outcomes (e.g., injuries) that may result from animal contact (3–6). Young infants may also be placed at increased risk of illness or injury if their caretakers are not cognizant of proper hygiene and safety procedures. These major life events might pose challenges to pregnant and postpartum women and their families, which might affect their relationship with the animals and might eventually lead to an unsuccessful pet ownership (7–11).

Health-care providers, such as physicians and veterinarians, play an important role in providing concerned individuals evidence-based information regarding animal contact and associated health outcomes. However, studies have found that the two professionals had very different views about the benefits and risks of animal-associated health outcomes (12–15) and they rarely consult with each other regarding the subject (14, 16–18). Furthermore, research has indicated that communication on this topic between clients and their health-care providers is poor (19–21). This lack of communication could severely impair public health education for this high-risk population, including pregnant women and new mothers. Studies have shown that an inadequate understanding of disease transmission might increase the risk of contracting infectious diseases from animal contact and that owners who are knowledgeable about potential risks and proper animal care are less likely to contract diseases from their animals (22, 23). To date, however, little is known about either the practices of pregnant women when they interact with companion animals or their perceptions of the associated health outcomes.

Here, we report the results of a study to assess the practices and perceptions of pregnant women and new mothers (up to 6 months postpartum) toward human–animal interactions and subsequent adverse health outcomes. The study was designed as the initial step to achieve the long-term goal of developing an evidence-based prevention program for pregnant women and new mothers to help them adopt certain behaviors to prevent adverse animal-associated health outcomes and enhance the bonds with their pets. More specifically, the study was conducted (1) to estimate dog and cat ownership in households with pregnant women and new mothers in the study areas and determine frequency and type of animal contact in the target population; (2) to assess pregnant women and new mothers’ perceptions and knowledge of animal-associated health outcomes; and (3) to identify information sources regarding animal contact and associated health outcomes that were known or sought by participants. To accomplish these aims, we used the Health Belief Model as the theoretical framework in developing the study survey targeting its five dimensions: perceived susceptibility, severity, benefits, barriers, and self-efficacy (24–26). The rationale for us to utilize this behavioral model was to gather data to assess the potential of an intervention on behavioral modification. For example, an intervention would have a greater potential if its target population perceives the importance of a problem (i.e., high perceived susceptibility and severity) and the benefits of an intervention and identifies fewer barriers to changing certain behaviors.

## Materials and Methods

### Study Sample and Study Sites

We recruited study participants through the women, infants, and children (WIC) programs in Champaign County, IL, USA (one clinic) and Tippecanoe County, IN, USA (two offices) in 2015. WIC is a nationwide program committed to improve the health of low-income pregnant and postpartum women, infants, and children by providing supplemental foods, health-care referrals, and education about nutrition and breastfeeding support. Women eligible for the study were 18 years and older and currently pregnant or with a newborn baby delivered within 6 months of the study. Researchers approached all the women who visited the WIC study sites and attended the breastfeeding and infant feeding classes without sampling to screen for eligibility and invited those who were eligible to participate. IRB exemption was granted for the study and only oral consent was obtained from participants. We planned to recruit 360 women based on an *a priori* sample size estimate^1^ that would allow us to achieve a margin error of ±5% at a confidence level of 95%, assuming that the true prevalence of pet ownership (for dogs or cats) in the source population was 30% (1) and adjusting for a 10% error rate in responses, which would include errors and missing values.

### Questionnaire Development and Measures

A paper-and-pencil questionnaire was developed to address each of the following study aims:

(1) Estimation of dog and cat ownership and animal contact: for the purpose of this study, dog and cat ownership was defined as currently having dogs or cats living in one’s household (including indoor and outdoor only pets). The survey included a question asking “How many dogs/cats currently live in your household?” followed by a series of questions about the characteristics of ownership and pet care practices, including age of animal, length of ownership, regular veterinary visits, and respondent’s attachment to animal [on a scale of 1 (very weak) to 5 (very strong)]. We also obtained information about the frequencies and types of animal contact, including contact with animals not owned. Information on animal-associated illness and injuries was also collected. Finally, we included questions about changes in ownership and animal-associated activities during or after pregnancy.
(2) Assessment of perceptions of animal-associated health outcomes: we include a set of statements about the perception of risks of disease transmission by animals and non-infectious animal-associated health outcomes, and changes in risk during pregnancy in the survey. These statements targeted the five dimensions of the Health Belief Model: perceived susceptibility, severity, benefits, barriers, and self-efficacy (24–26). Participants were asked to rank how strongly they disagreed or agreed with each of the statements using a five-point Likert Scale with 1 indicating strongly disagree, 2 indicating disagree, 3 indicating neutral, 4 indicating agree, and 5 indicating strongly agree. The questions were further grouped into four categories: susceptibility and severity (*n* = 5), benefits (*n* = 5), barriers (*n* = 4), and self-efficacy (*n* = 6). The design and classification of these questions were pre-determined by investigators (Table 1). Cronbach’s alpha was used to assess internal consistency across the statements within each of the four groups pertaining the five dimensions of the Health Belief Model.
(3) Identification of information sources: we were particularly interested in participants’ perceptions of the role of human health-care providers and veterinarians as sources of information about animal contact and associated health outcomes. Other potential information sources investigated in this study include television, friends and family, print media, and the Internet.

**Table 1:** **Perceptions toward animal contact and associated health outcomes in pregnant women and new mothers**.

Statement	Strongly disagree (1) to strongly agree (5)					Mean
	1	2	3	4	5	
**Susceptibility and severity (*n*** **= 318, mean = 3.4)**						
There are diseases that I can get from my pets and other animals even if they appear healthy	16 (5)	16 (5)	58 (18)	145 (46)	83 (26)	3.8
Pregnancy makes me more prone to getting diseases from animals and may make the disease worse if I do get it	20 (6)	45 (14)	70 (22)	115 (36)	68 (21)	3.5
Young infants are more prone to getting diseases from animals than older children and may have a worse illness than an older child if they do get sick	11 (4)	27 (9)	56 (18)	129 (41)	95 (30)	3.9
I worry about getting a disease or being injured by an animal during my pregnancy	61 (19)	79 (25)	74 (24)	50 (16)	51 (16)	2.8
I worry about my infant or unborn child contracting a disease or being injured by an animal	51 (16)	58 (19)	76 (24)	61 (20)	67 (21)	3.1
**Benefits (*n*** **= 319, mean = 4.2)**						
Using gloves and washing my hands during and after handling animal waste will help keep me and my unborn child or young infant healthy	13 (4)	6 (2)	37 (12)	93 (29)	167 (53)	4.3
Using care to avoid being injured by animals will help keep me and my unborn child or young infant healthy	11 (4)	5 (2)	31 (10)	125 (39)	145 (46)	4.2
It is important for young infants to always be closely supervised around animals to prevent injuries even if animals seem to be friendly	9 (3)	8 (3)	17 (5)	87 (27)	197 (62)	4.4
It is important for me to be very careful around animals during pregnancy because I am more prone to being accidently injured due to falling or lifting things that were not too heavy for me in the past	14 (4)	15 (5)	39 (12)	128 (40)	122 (38)	4.0
Adopting hygiene measures is important for keeping me and my family healthy	13 (4)	5 (2)	32 (10)	113 (36)	154 (49)	4.2
**Barriers (*n*** **= 326, mean = 2.2)**						
Adopting hygiene measures such as washing hands or wearing gloves during and after handling animal waste is difficult	106 (34)	100 (32)	45 (14)	42 (13)	22 (7)	2.3
Adopting hygiene measures will make spending time with animals less fun	114 (36)	112 (36)	51 (16)	34 (11)	4 (1)	2.1
Not allowing animals near me while pregnant or my young infant in order to prevent injuries is difficult	53 (17)	98 (31)	77 (24)	62 (20)	26 (8)	2.7
Having animals regularly examined by a veterinarian is unnecessary if they appear healthy to me	126 (40)	104 (33)	42 (13)	31 (10)	12 (4)	2.0
**Self-efficacy (*n*** **= 319, mean = 4.1)**						
In general, I am more careful with hygiene around animals now that I am pregnant/having a young infant	15 (5)	11 (4)	56 (18)	107 (34)	129 (41)	4.0
In general, I am more careful to avoid being injured (or have my child be injured) when interacting with animals now that I am pregnant/having a young infant	9 (3)	8 (3)	37 (12)	120 (38)	141 (45)	4.2
I know what things to do to keep myself and my family healthy and safe around animals	11 (4)	15 (5)	51 (16)	137 (43)	102 (32)	4.0
I know where to find information about keeping myself and my family healthy and safe around animals	10 (3)	10 (3)	44 (14)	138 (44)	111 (36)	4.1
I feel comfortable asking veterinarian relevant information	7 (2)	6 (2)	48 (15)	130 (41)	126 (40)	4.1
I feel comfortable asking doctor relevant information	9 (3)	2 (0.6)	21 (7)	139 (44)	146 (46)	4.3

*Participants were asked to rank (from 1 to 5) how strongly they disagreed or agreed with each statement. The statements are grouped by the five dimensions (i.e., perceived susceptibility, severity, benefits, barriers, and self-efficacy) of Health Belief Model. Frequencies (%) and mean scores are present*.

The questionnaire was designed to be completed in approximately 15 min by participants with sixth grade reading level. In order to reduce time and efforts for participants to complete the survey, most questions were designed in a multiple-choice format, and series questions were present in a tabular format with the same corresponding answers. For example, all questions regarding frequency of dog contact were present in a table with the same choices of “daily,” “more than once a week but less than daily,” “about once a week,” “less than once a week,” and “never” in the same order. The questionnaire was pilot tested by 10 eligible women who participated in the WIC program in Champaign County, Illinois and was revised accordingly.

### Statistical Analysis

Estimates of the prevalence of dog and cat ownership were derived and compared using mixed generalized linear models. Individual participant was included as a random effect in the mixed models to account for dependency for survey respondents who owned both dogs and cats. We also used mixed generalized linear models to compare frequencies of and changes in animal contact practices during and after pregnancy between dog and cat owners. We used Student’s *t*-test to compare mean knowledge/perception scores between pet owners and respondents who did not have pets, and between dog and cat owners, excluding those who owned both dogs and cats. A *p*–value <0.05 was considered statistically significant, and we used IBM SPSS Statistics for Windows (version 22.0, IBM Corp., Armonk, NY, USA) to perform statistical analyses.

## Results

A total of 326 eligible women were surveyed during the study period. Among them, 253 were recruited from the two WIC offices in Tippecanoe County, IN, USA and 73 from the WIC clinic in Champaign County, IL, USA. The overall response rate was 91%, with a lower response rate (68%) at the Illinois study site. Most of the respondents were between 18 and 34 years old (90%) with the highest education level being high school or some college (65%). The majority of participants were non-Hispanic white (56%) followed by African-American (21%).

### Dog and Cat Ownership, Animal Contact, and Associated Health Outcomes

Overall, 45% (95% CI: 39–51%) of the participants reported having at least one dog or one cat in their households. The prevalence of dog ownership at 39% (95% CI: 33–45%) was significantly higher (*p* = 0.001) than cat ownership at 26% (95% CI: 21–31%). Among pet owners, 76% rated their attachment to their pets as strong or very strong. Furthermore, 18% felt that their attachment to the pets became stronger during or after pregnancy, while 24% felt that their attachment became weaker. Among the survey respondents, 6 and 3% reported ending a relationship with a dog or a cat during and after their pregnancies, respectively.

**Figure 1:**
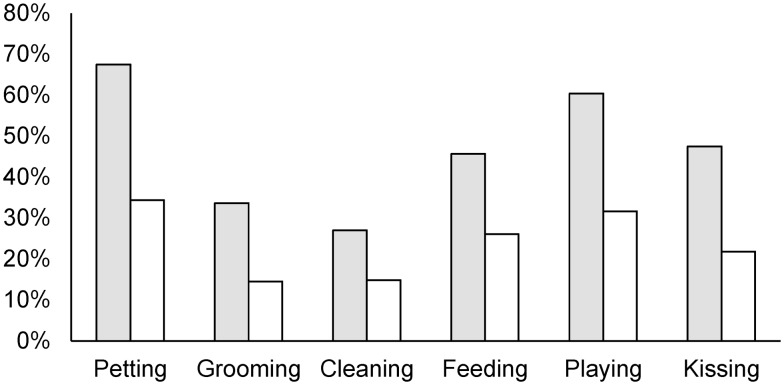
**Proportion of respondents having different types of dog and cat contact in the month prior to the study**. Gray bars are for dog contact and white bars are for cat contact.

**Figure 2:**
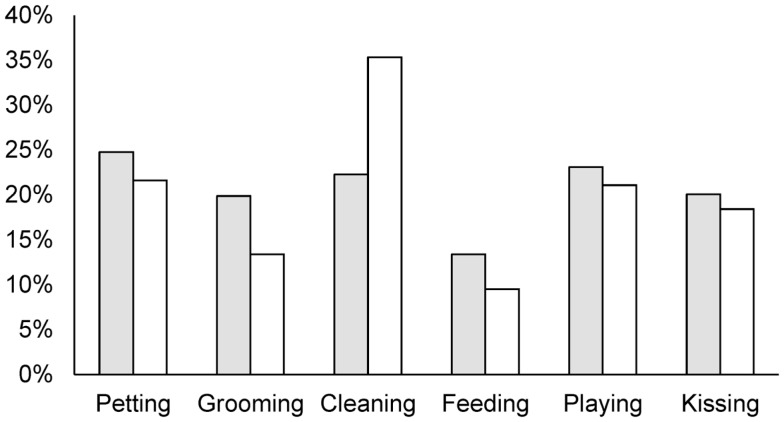
**Proportion of respondents discontinuing or decreasing dog and cat contact during or after pregnancy**. Gray bars are for dog contact and white bars are for cat contact.

Regardless of pet ownership, 74% of the study participants reported having some type of contact with dogs or cats in the month prior to the study. The percent of participants with dog contact (69%) was significantly higher (*p* < 0.001) than those with cat contact (34%). Forty-eight percent of the participants reported daily contact with dogs or cats in the month prior to the study. The percent of daily contact with dogs (40%) was also significantly higher (*p* < 0.001) than daily contact with cats (20%). Among those who walked dogs throughout the month prior to the study (*n* = 74), the reported median (range) time (minutes per day) spent dog walking was 20 (3–160). Among dog walkers, 38% reported discontinued or decreased dog walking activities during and after their pregnancies. Figure 1 shows the animal contact activities reported by the respondents. The respondents consistently reported higher levels of dog contact compared to cat contact across different activities. Figure 2 shows the level of discontinued or decreased animal contact activities during or after pregnancy among those who reported having animal contact in the month prior to the study. The reported levels were comparable between dog and cat contact except for cleaning, where we noted a higher proportion of discontinuation and decrease for cats.

Eighty-seven percent of the respondents reported washing their hands after cleaning the feces of a dog most of the time or always compared to 73% reported after cleaning cat litter (*p* = 0.019). During and after pregnancy, 25 and 14% of the respondents reported increasing the frequency of hand washing after dog and cat contact, respectively. Among dog owners, 72% took their dogs to a veterinarian at least once a year, while 25% reported no regular veterinary visits. Among cat owners, 49% took their cats to a veterinarian at least once a year, and 49% reported no regular veterinary visits. Regular veterinary visits were significantly different (*p* < 0.001) between dog and cat owners. Among all respondents, 14% agreed or strongly agreed that having animals regularly examined by a veterinarian is unnecessary if they appear healthy (Table 1). Eleven respondents (4%) or their household members had been diagnosed with a non-injury illness that was transferred from animals. Overall, 42% of respondents reported having some type of animal-associated injuries, and 56% of the injuries were caused by animals the respondents owned. Scratches (37%) were the most frequently reported injury followed by bites (18%).

### Perceptions of Animal-Associated Health Outcomes

The respondents’ perceptions of animal contact and associated health outcomes during and after pregnancy are summarized in Table 1, grouped by the five dimensions of the Health Belief Model. The Cronbach’s alphas ranged from 0.70 for perceived barriers to 0.85 for perceived benefits and self-efficacy. Overall, the perceived benefits and self-efficacy categories received the highest mean score (>4), while the perceived barriers category received the lowest mean score (2.3). Pet owners were more likely to agree with the statement that avoiding animals for disease prevention was difficult compared with respondents who did not have a pet (*p* = 0.007). Pet owners were also more likely to agree or strongly agree that they have knowledge about keeping themselves and their families healthy and safe around animals (*p* = 0.015). By contrast, pet owners were less likely to worry about contracting a disease or being injured by an animal personally (*p* = 0.042) and about their infants or unborn children contracting a disease or being injured (*p* = 0.008) than respondents who did not have a pet. Cat owners were more worried about contracting a disease or being injured by an animal personally and about their infants or unborn children contracting a disease (*p* = 0.031) or being injured (*p* = 0.048) than dog owners. In summary, pet owners had a higher mean score (4.2) for the perceived self-efficacy category than those who did not have pets (4.0), while cat owners had a higher mean score (3.6) for perceived susceptibility and severity than dog owners (3.2).

### Information Sources

**Figure 3:**
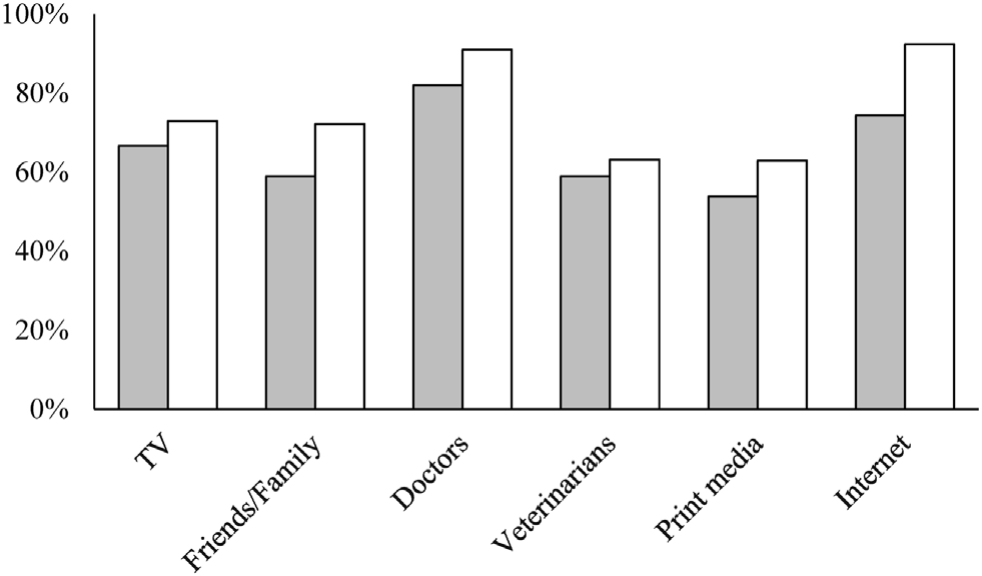
**Sources for which participants would seek information on animal contact and associated health outcomes**. Gray bars are for pet owners and white bars are for non-pet owners.

Eighty percent of the survey respondents reported that they know where to find information about prevention of animal-associated illness and injuries. Pet owners were more confident in their knowledge of where to find information about animal contact and associated health outcomes than respondents who did not have pets (*p* = 0.002). Nonetheless, the two groups were similar in identifying sources from which participants would seek information about animal contact and associated health outcomes (Figure 3). Human health-care providers and the Internet were the most frequently reported information sources. The majority of participants felt comfortable asking health-care providers (90%) and veterinarians (81%) about prevention of animal-associated illness and injuries. Pet owners had a higher mean score (4.3), which indicates a higher level of agreement with the statement that they feel comfortable asking veterinarians relevant questions than respondents who did not have pets (*p* = 0.027). Cat owners also showed a higher mean score than dog owners in responding to this question (*p* = 0.030). In addition, 75 and 72% of the respondents agreed or strongly agreed that health-care providers and veterinarians, respectively, should initiate discussions about preventing animal-associated illness and injuries. However, among those who had visited doctors or prenatal care services, only 41% reported that their health-care providers discussed animal-associated illness and injuries. Similarly, 41% of respondents who had visited veterinarians reported that the veterinarian discussed these issues.

## Discussion

We conducted this study to collect baseline data on the practices and perceptions of pregnant women and new mothers (up to 6 months postpartum) toward animal contact and associated health outcomes. Our long-term goal is to develop an evidence-based intervention for this target population to help them adopt certain behaviors to prevent animal-associated adverse health outcomes and enhance the bond between owners and their pets. We expect the implementation of such an intervention to benefit both human health and the well-being of animals. The behavior changes that will occur during pregnancy as a result of this intervention are also expected to positively influence the early lives of infants. The results from this study provide scientific evidence to support the development of an intervention and potential implications of program implementation.

The percentage of households that owned dogs (39%) and cats (26%) estimated in our study were comparable to the estimates derived from a nationwide United States survey (1). The majority of pet owners in this study also reported a strong attachment to their pets, similar to the results of the AVMA survey (1). The low incidence of discontinued pet ownership (6% for dogs and 3% for cats) found in this study does not directly support the notion that pregnancy or the birth of a child as a risk factor of pet relinquishment (7–11). However, we cautioned readers that we did not include a comparison group in the study and further epidemiological studies are warranted to assess whether pregnancy or the birth of a child is associated with pet relinquishment. Animal contact was prevalent (74%) among the study participants. Pregnancy or the birth of a child altered some animal contact practices among the study participants. Particularly, 35% of respondents discontinued or decreased the activity of cleaning cat litter boxes during and after their pregnancies (Figure 2). This greater change in cat contact and the finding that cat owners worried more than dog owners about contracting a disease from animals support the idea that pregnant women might be more aware of the diseases that can be transmitted by cats than the diseases that can be transmitted by dogs. However, reports of diseases contracted from animals were low (4%) in this study. By contrast, animal-associated injuries were prevalent (42%), and the majority were caused by animals the respondents owned (56%). The finding that the majority of animal-associated injuries were caused by owned dogs and cats was also reported in other studies (27, 28). Although we did not assess women’s knowledge of specific zoonotic diseases and animal-associated injuries, these findings suggest the importance of providing accurate information about preventing animal-associated health outcomes to pet owners.

We included survey questions targeting the five dimensions of Health Belief Model in order to collect data to investigate the potential of an intervention for pregnant women and new mothers to help them adopt certain behaviors to prevent adverse animal-associated health outcomes and enhance the bonds with their pets. Among the five dimensions, the perceived benefits and self-efficacy categories received the highest mean score, while the perceived barriers category received the lowest mean score. These findings are encouraging because they suggest that the target population appreciates the potential benefits of a relevant prevention program and does not strongly resist changing certain behaviors. We also observed some differences in the responses between pet owners and those who do not have pets, as well as between dog and cat owners. Overall, pet owners had a higher mean score for the perceived self-efficacy category than those who did not have pets, while cat owners had a higher mean score for perceived susceptibility and severity than dog owners. These differences indicate potential heterogeneity within the target population with respect to pet ownership. Therefore, different intervention programs to target pet and non-pet owners might be more efficient than a uniform program across the two populations. Finally, we noted that the classification of questions was pre-determined rather than data driven and we did not validate them with respect to the appropriateness of measuring the five dimensions of Health Belief Model. However, we performed Cronbach’s alpha to assess internal consistency among the questions within each group. The results indicated an acceptable to good internal consistency (i.e., alphas ranged from 0.70 to 0.85). Furthermore, we presented the relevant questions in Table 1 so that other interested investigators could cross-examine them using different data.

Identification of relevant information sources for which the target population would seek is essential to plan the dissemination of a prevention program. As shown in Figure 3, non-pet owners consistently reported a higher proportion across information source categories than pet owners. This observation could be explained by the finding that pet owners were more confident in their knowledge of animal contact and associated health outcomes. The study found that most respondents identified doctors and prenatal care providers as sources of relevant information. The study participants also, in general, recognized and expected them to provide such information. By contrast, veterinarians were less recognized as an information source compared to human health-care providers, and this gap between human health-care providers and veterinarians remains even among pet owners. Promoting the role of veterinarians in discussing animal-associated health outcomes with clients is important because they typically receive more training in zoonotic diseases and proper animal handling than human health-care providers (29). The differences in training and practice between the two professionals might further explain the disparities in their knowledge of and perceptions toward animal contact and the associated health outcomes observed in previous studies (12–15). The One Health initiative provides an opportunity to bridge the knowledge gap between human medicine and veterinary medicine (16, 30, 31). The One Health concepts are built on the idea that human health and animal health are closely connected through a shared environment. The relationship between humans and animals is complex and could have various beneficial and adverse effects on human health as well as animal health within a shared social context (32, 33). We will apply the One Health approach to the development of a prevention program, focusing not only on the prevention of zoonotic diseases but also injuries (e.g., bites and scratches) and non-infectious health outcomes (e.g., allergies) in pregnant women, new mothers, and their young infants.

The women in this study were recruited through WIC programs. While WIC programs provide us easy access to the population of interest, these programs cover only a selective subset of pregnant women and new mothers (i.e., those who are from low-income families). This non-random sampling from a limited number of study sites may constrain the generalizability of the findings derived from the study. African-Americans were overrepresented in this study compared to the racial composition in Tippecanoe and Champaign Counties. Other demographic data on the study participants are described in the results. During the recruitment period, we did not achieve the planned sample size of 360 participants. However, the estimated prevalence of dog and cat ownership was comparable with estimates derived from a nationwide survey, and the associated 95% confidence intervals indicate an acceptable precision for these estimates. Because only <40% of the respondents provided valid answers to the questions asking about their due date and/or the age of a child, we were not able to perform further analyses based on their current pregnancy status.

In summary, the prevalence of dog and cat ownership in the households with pregnant women and new mothers derived from this study was comparable to the national estimate in the United States and animal contact was found to be prevalent among the study participants. Pregnancy or the birth of a child altered some animal contact practices among the study participants; particularly a discontinuation or decrease in cleaning cat litter boxes. Reports of diseases contracted from animals were low (4%) in this study. By contrast, animal-associated injuries were prevalent (42%), and the majority were caused by animals the respondents owned (56%). The study findings also suggest that the target population appreciates the potential benefits of a prevention program concerning animal contact and associated health outcomes and does not strongly resist changing certain behaviors. Finally, veterinarians were less recognized as an information source for animal contact and associated health outcomes compared to human health-care providers among the study participants. These results together provide scientific evidence for developing an intervention to help pregnant women and new mothers have healthier and safer animal contact practices.

## Author Contributions

H-YW designed the study, analyzed and interpreted the data, and drafted and finalized the manuscript. KA reviewed and assisted in developing the study survey, set up study sites, conducted the survey, and reviewed the manuscript.

## Conflict of Interest Statement

The authors declare that the research was conducted in the absence of any commercial or financial relationships that could be construed as a potential conflict of interest.

## Footnotes

^1^Sample size calculation was performed using Epi Info™ (version 7, Centers for Disease Control and Prevention, Atlanta, GA, USA).
